# Practical Departments

**Published:** 1901-11-16

**Authors:** 


### PRACTICAL DEPARTMENTS.

### RAILWAY LAVATORIES.

The provision of lavatory accommodation on English railway trains has become so much more common in the last few years, that some consideration of the construction of the lavatories, from a sanitary point of view, may not be out of place. It is not a little curious to find these modern contrivances have most of the defects which have long ago been exposed and dealt with in our homes, and even in our ships. There are two usual arrangements—the lavatory compartment and the corridor lavatory. The Pullman car has in effect a third, but it is really a variation of the second. Of the two first, the lavatory compartment is by far the most dangerous to health, if improperly constructed. The lavatory, usually ill-ventilated itself, is in direct connection with a compartment holding from five to nine persons, already far too small to give sufficient cubic space for these persons for any length of time. Any foul air from the lavatory must be breathed by the occupants of the compartment during a period which may last from two to 10 hours, or even more. In corridor trains and in Pullman cars, the lavatory, though often badly ventilated, opens into a more or less airy corridor, and its power for injury is much more limited.

The fittings of the lavatory usually consist of a closet and wash-hand basin, with the addition of a water-bottle and glass for drinking purposes. The window is very rarely made to open, but some lines have ventilators which produce a good current of air when the train is in motion. The water-bottle has no stopper, so that its contents are exposed to the air of the lavatory. The closet is usually a simple hopper basin, without a trap, but sometimes there is a flap on the outlet, while the seat has generally a wooden lid. There is a supply of water from a cistern above, which also supplies the lavatory basin. The flush is usually quite insufficient to cleanse the basin and outlet pipe, and the arrangement is, at best, no better than a trapless water-closet, with an unventilated soil pipe, while, if left without water, as is frequently the case, it closely resembles a common privy. Neither of these systems should be permitted for a convenience directly attached to what is, in effect, a sitting-room. The strong upward draught, with a most offensive smell, which is always observable passing through the basin when the train is moving, is a serious danger to health, which is greatly increased when the lid to the closet seat is omitted.

The wash-hand basin takes various forms, the best being the fixed basin with marble top, found on the corridor trains, and the worst the tip-up basin discharging into the closet, found in some lavatory compartments on the southern lines. In the latter case the basin is placed over the closet; the latter has no lid, and it is impossible to wash one's hands without breathing the foul air passing up from the basin referred to above. The wash-hand basins are usually untrapped. This is a matter of some importance in the lavatory compartment, but not so, perhaps, in the corridor lavatory, as the waste pipe is short.

It is not probable that a sanitary authority, however willing, could enforce any existing bye-laws upon the owners of a train passing through its district, and it may be that legislation will be necessary to secure any improvement. One may hope, however, that if the attention of the great railway companies were called to the matter they would, for their own credit's sake, take steps to ensure that the following conditions, at least, should be observed in constructing these conveniences:—

1. The lavatory should always be approached by a ventilated corridor, or a short ventilated lobby. Lavatories opening directly from an ordinary compartment should be entirely abolished.

2. The W.C. should be trapped, as in the patterns now usual on ships. Existing closets having no lids should be fitted with them without delay.

3. The outlet pipe from the closet should be carried up full bore to the roof of the carriage, so that the air drawn through it will pass out at the top and not into the compartment.

4. The cisterns should be automatically filled by a pump on the engine or some other suitable contrivance, so as not to leave the water supply to over-worked porters.

5. The wash-hand basins should have a trapped waste pipe, or one that is closed by the shutting of the basin in the folding patterns. The waste pipe should have no connection with the closet and the basin should never be placed over the latter.

6. The windows of the lavatory should always be made to open freely, or full ventilation should be secured by other means, independently of the door.

7. The water-bottle (if supplied) should have a glass stopper and be filled with water from a suitable source, under responsible supervision,

